# Nephrology Trainee Education Needs Assessment: Five Years and a Pandemic Later

**DOI:** 10.1016/j.xkme.2022.100548

**Published:** 2022-09-30

**Authors:** Benjamin S. Ko, Kurtis A. Pivert, Rob Rope, Anna M. Burgner, Joshua S. Waitzman, Susan M. Halbach, Suzanne M. Boyle, Lili Chan, Stephen M. Sozio

**Affiliations:** 1 University of Chicago, Chicago, Illinois; 2 American Society of Nephrology, Washington, District of Columbia; 3 Oregon Health & Science University, Portland, Oregon; 4 Vanderbilt University Medical Center, Nashville, Tennessee; 5 Beth Israel Deaconess Medical Center, Boston, Massachusetts; 6 Seattle Children's Hospital, Seattle, Washington; 7 Temple University, Philadelphia, Pennsylvania; 8 Icahn School of Medicine at Mount Sinai, New York, New York; 9 Johns Hopkins University School of Medicine and Bloomberg School of Public Health, Baltimore, Maryland

To The Editor:

Seventy-four percent of nephrology fellows reported exclusively receiving virtual didactics during the early part of the coronavirus disease 2019 (COVID-19) pandemic.^1^ In response to this rapid shift to online instruction, we sought to describe usage of novel digital educational resources, including free open access medical education (FOAMed; eg, online journal clubs, podcasts, and blogs), by current adult and pediatric nephrology fellows through a research survey. Observed utilization rates were compared with rates reported from a prepandemic study.^2^

Our cross-sectional survey targeted 920 current adult and pediatric nephrology fellows. Participants gave informed consent by clicking a checkbox after reading the study information. Questions assessing fellows’ educational resource usage, fellows’ perceived effectiveness of each resource they utilized, and fellows’ overall rating of the education quality in their programs were drawn from a 2016 cross-sectional survey of adult trainees (320 participants, 37% response) previously tested for face and content validity (Item S1).^2^ Our survey was disseminated via email May 4 to 31, 2021, using unique uniform resource locators (Qualtrics) (Item S2).

Educational resources were categorized as traditional (eg, textbooks), digital-paid (eg, UpToDate), or digital-FOAMed (eg, NephJC). Intracohort differences were analyzed based on respondents’ medical school (international medical graduates vs US medical graduates) and median respondent age (less than or equal to 33 years vs greater than or equal to 34 years). Changes between 2016 and 2021 responses were also assessed (χ^2^ tests, GraphPad Prism). As respondents could skip questions, data were not imputed, and per-question percentages were calculated. Identifiers were separated from responses, and both were securely stored (Johns Hopkins institutional review board #00205206).

Five hundred one fellows, representative of trainees by sex (adult fellows) and year of training, participated in 2021 (54% response) (Table S1). UpToDate remained the most used (84%) and highly rated (66%, “very effective”) resource (Table 1). Most participants rated the quality of their education highly (83%, “good”/“excellent”), believed their education was sustained during the pandemic (83%), and self-assessed as prepared for independent practice upon graduation (87%).

**Table 1:** Educational Resource Rankings by Cohort

Resource (N Using Resource)	All Fellow Respondents	Resource Used by Fellows Stratified by Educational Status		Resource Used by Fellows Stratified by Fellow Age (Median Age = 33 y)	
	% Respondents Rated Very Effective	IMGs, % (n)	USMGs, % (n)	Age ≤33 y, % (n)	Age ≥34 y, % (n)
**Digital-Paid**^a^					
UpToDate **(419)**	66%	100% (223)	100% (196)	100% (240)	100% (172)
ASN KSAP **(293)**	65%	69% (154)	71% (139)	67% (161)	73% (126)
ASN NephSAP **(191)**	45%	48% (108)	42% (83)	45% (108)	47% (81)
Doximity **(43)**	18%	12% (26)	9% (17)	10% (23)	12% (20)
**Digital-FOAMed**^b^					
Renal Fellow Network **(235)**	40%	52% (117)	60% (118)	59% (141)	52% (90)
NephJC **(166)**	46%	34% (76)	46% (90)	45% (107)	34% (58)
Twitter **(145)**	35%	30% (67)	40% (78)	40% (97)	27% (46)
NephMadness **(144)**	31%	29% (64)	41% (80)	38% (91)	29% (50)
Nephrology podcasts **(118)**	38%	28% (62)	29% (56)	31% (75)	24% (41)
GlomCon **(106)**	56%	27% (60)	23% (46)	26% (62)	25% (43)
NephSIM **(98)**	58%	25% (55)	22% (43)	23% (56)	24% (42)
*AJKD* blog **(77)**	41%	19% (42)	18% (35)	20% (47)	17% (29)
Arkana Pathology Series **(66)**	39%	18% (40)	13% (26)	18% (42)	14% (24)
NephroPOCUS **(44)**	31%	11% (24)	10% (20)	11% (26)	10% (17)
Medicine podcasts **(43)**	35%	9% (19)	12% (24)	12% (28)	8% (14)
**Traditional**					
*CJASN* articles **(274)**	39%	58% (130)	73% (144)	69% (165)	62% (106)
KDIGO/KDOQI Clinical Practice Guidelines **(255)**	42%	61% (137)	60% (118)	61% (146)	62% (107)
*JASN* articles **(249)**	33%	56% (124)	64% (125)	59% (142)	60% (104)
*AJKD* articles **(222)**	37%	47% (104)	60% (118)	55% (133)	49% (85)
Textbooks **(220)**	37%	52% (115)	54% (105)	48% (115)	59% (101)
Journal articles in general **(119)**	30%	36% (80)	61% (119)	50% (119)	45% (77)

Abbreviations: *AJKD*, *American Journal of Kidney Diseases*; ASN, American Society of Nephrology; *CJASN*, *Clinical Journal of the American Society of Nephrology*; FOAMed, free open access medical education; IMG, international medical graduate; *JASN*, *Journal of the American Society of Nephrology*; KDIGO, Kidney Disease: Improving Global Outcomes; KDOQI, Kidney Disease Outcomes Quality Initiative; KSAP, Kidney Self-Assessment Program; USMG, US medical graduate.

a ASN KSAP and ASN NephSAP are free for ASN members. Nephrology fellows are eligible for complimentary ASN membership during training.

b One nephrology fellow indicated using Sermo.

US medical graduates and international medical graduates used traditional resources similarly, yet more US medical graduates utilized digital resources (Twitter: 40% vs 30% for international medical graduates; NephJC: 46% vs. 35%; and NephMadness: 42% vs. 29%; *P* < 0.05 for all). Traditional resource use was similar between age cohorts—less than or equal to 33 and greater than or equal to 34 years—yet FOAMed digital resource use was significantly higher in the younger cohort (Twitter: 41% vs 26%; NephJC: 47% vs 34%; nephrology podcasts: 32% vs 23%; and NephMadness: 39% vs 28%; *P* < 0.05 for all).

Between 2016 and 2021, digital resource use generally increased (Fig 1) with more fellows using NephJC (from 7% to 32%; 46% rating it “very effective” in 2021) and the Kidney Self-Assessment Program (27% to 58%; 65% “very effective” in 2021). Among new resources, NephMadness was the most used (28%), and NephSIM and GlomCon the highest rated (58% and 56%, “very effective,” respectively). NephJC, NephSIM, and GlomCon were perceived as “very effective” by >50% of users, similar to another study of NephSIM users.^3^

**Figure 1:**
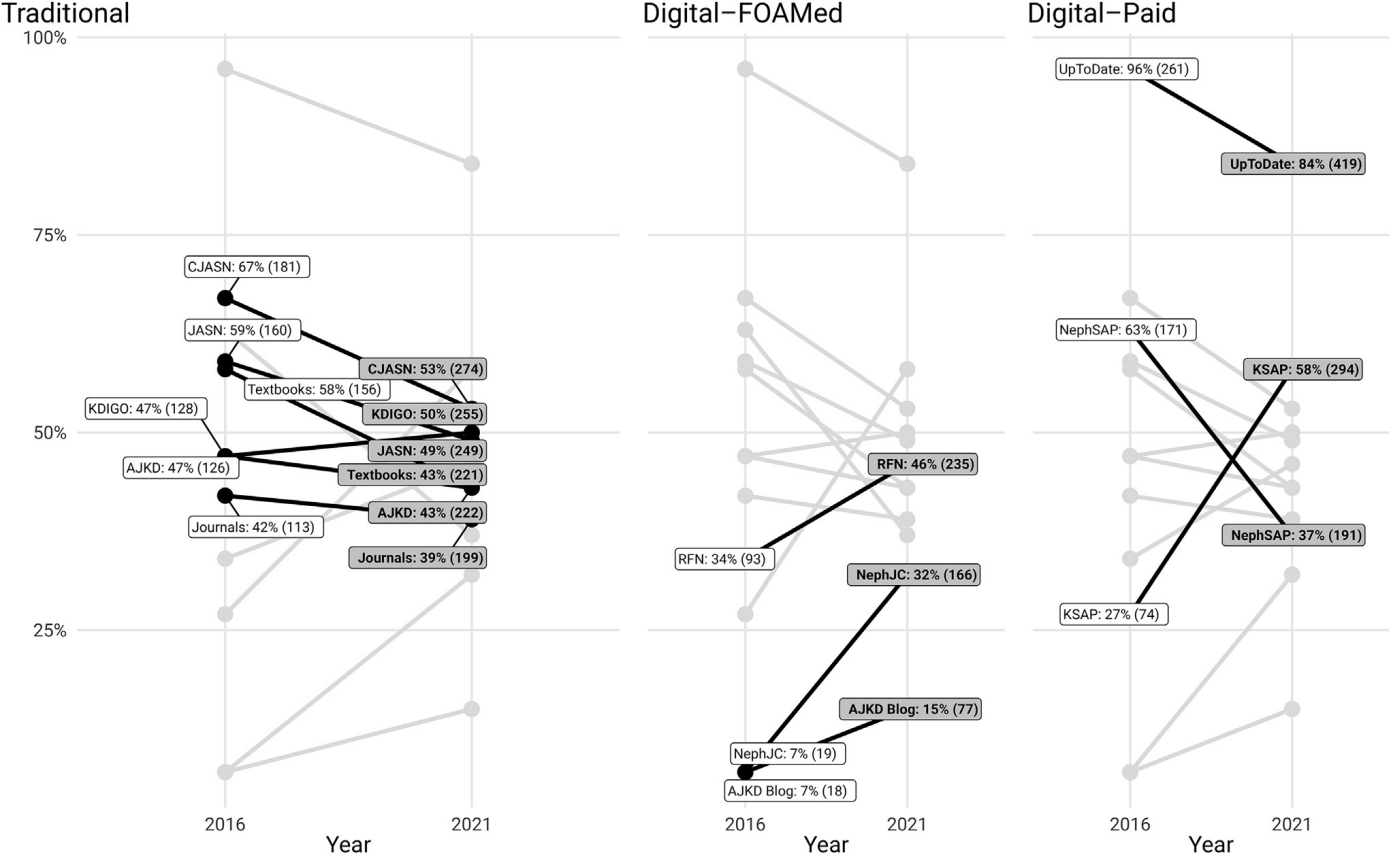
Change in educational resource use between 2016 and 2021 by resource type. Data shown as percentages (number of respondents) for each cohort: 2016, 320 respondents, response rate 37%; 2021, 501 respondents, response rate 55%. KSAP and NephSAP are free for ASN members, and nephrology fellows are eligible for complimentary ASN membership during training. Abbreviations: *AJKD*, *American Journal of Kidney Diseases*; *CJASN*, *Clinical Journal of the American Society of Nephrology*; *JASN*, *Journal of the American Society of Nephrology*; KDIGO, Kidney Disease: Improving Global Outcomes; KSAP, Kidney Self-Assessment Program; NephJC, Nephrology Journal Club; NephSAP, Nephrology Self-Assessment Program; RFN, Renal Fellow Network.

These trends are not unique to nephrology. A 2017 study of residency program directors reported widespread use of “asynchronous, e-learning” content, with 71% of residencies using digital resources at least sometimes or more^4^ with perceived-effectiveness ratings comparable to our study. Meta-analyses demonstrated asynchronous resources improved educational outcomes comparably to traditional ones^5^ and found “blended” resources (synchronous and asynchronous) noninferior to traditional ones.^6^ Substituting 1 hour of traditional lectures with asynchronous digital content in an emergency medicine program resulted in no change in in-training examination scores.^7^ This suggests digital learning is at least noninferior to traditional methods and offers advantages like flexible scheduling.

Our study has important limitations. We did not assess how frequently fellows use each resource or which were most preferred. We were also unable to measure the impact of these resources on outcomes, such as board scores and patient safety, and note that cohort stratification variables may not be substantively meaningful. Despite these limitations, our description of educational resource use provides insight into nephrology fellows’ learning habits, which can inform curricular development.

Our findings underscore the need for critical appraisal of quality of novel digital resources like FOAMed. Future studies should focus on (1) applying validated tools to assess FOAMed content and reliability and (2) measuring hard outcomes (eg, certification examination scores) among users and nonusers.^8^ Because FOAMed is universally available, it represents an opportunity to provide standardized education to training programs regardless of size or location. However, our data suggests that international medical graduates and older fellows use digital resources less frequently. Therefore, if future studies demonstrate that digital resources associate with positive outcomes, work is needed to ensure that all faculty and fellows are able to access and interface with them.

Strengths of our study include a nationally representative sample of nephrology fellows with a high response rate and a comparison between fellow responses between 2016 and 2021.

In conclusion, digital education resources use significantly increased between 2016 and 2021. Studies are needed to evaluate their quality and effectiveness relative to more traditional ones.

## Supplementary Material

Supplementary File (PDF) Item S1-S2, Table S1.
