# Supplementary material for: Nephrology Trainee Education Needs Assessment: Five Years and a Pandemic Later
Source: Kidney Med. 2022 Sep 30;4(11):100548. doi: 10.1016/j.xkme.2022.100548 (PMC9575331; doi:10.1016/j.xkme.2022.100548)
Supplement: Supplementary File (PDF) — Item S1-S2, Table S1. [file mmc1.pdf]

Item S1: 2016 ASN Nephrology Fellow Survey Instrument

1. ELECTRONIC CONSENT: Please select your choice below.
Clicking on the 'Agree' button below indicates that:
You have read the above information
You voluntarily agree to participate
You are at least 18 years of age

- Yes
- No

2. Please select your fellowship program.

3. Are you in a pediatric nephrology fellowship?

4. What is your gender?

5. How old are you?

6. What is your current citizenship status?

7. What was your citizenship status when you began your nephrology training?

8. What is your race? (Select all that apply.)

- American Indian/Alaska Native
- Asian or Pacific Islander
- Black/African American
- White
- Other

9. Please specify.

10. Are you Hispanic/Latino?

11. Where did you live when you completed your secondary education (i.e., high school)?

12. Please specify.

13. What type of medical education do you have?

14. Where did you attend medical school?

15. Please specify.

16. In what state did you do your internal medicine training?

17. At the end of the 2015-2016 training year how many total years of nephrology training will you have completed?

18. What is your current level of educational debt?

19. Do you have an obligation or visa requirement to work in a federally designated Health Professional Shortage Area?

20. What do you expect to be doing at the end of the 2015-2016 training year? (Select one.)
21. Please specify.

22. What additional training are you pursuing? (Select one.)

23. Please specify.

24. Are you planning to stay in your state of training for your first nephrology job?

25. Have you searched for a nephrology job beginning after the 2015-2016 training year?

26. Have you been offered a nephrology job beginning after the 2015-2016 training year?

27. Did you have difficulty finding a nephrology job you were satisfied with?

28. What would you say was the main reason you had difficulty? (Select one.)

29. Please specify.

30. Did you have to change your plans because of limited nephrology job opportunities?

31. How many jobs did you apply for (excluding fellowships and other training positions)?

32. How many job offers did you receive (excluding fellowships and other training positions)?

33. Were there certain types of nephrology jobs or settings that were more available than others?

34. Please describe.

35. Were there certain types of nephrology jobs or settings that were less available than others?

36. Please describe.

37. Which of the following best describes the focus of your primary nephrology job? (Select one.)

38. Which of the following best describes the type of practice setting of your primary nephrology job?

39. Please specify.

40. Which of the following describes your responsibilities in your primary nephrology job? (Select all that apply.)

- Hospital care
- Outpatient nephrology care
- Diagnostic ultrasonography
- Kidney biopsy
- Temporary dialysis catheter placement
- Interventional nephrology
- Joint venture with a dialysis provider
- Medical directorship with a dialysis provider
- Basic science research
- Clinical research

- Other

41. Please specify.

42. Outside of your primary nephrology job responsibilities do you expect to hold a secondary nephrology job (e.g., moonlighting medical directorship)?

43. Which of the following describes your responsibilities in your secondary nephrology job(s)? (Select all that apply.)

- Hospital care
- Outpatient nephrology care
- Diagnostic ultrasonography
- Kidney biopsy
- Temporary dialysis catheter placement
- Interventional nephrology
- Moonlighting (nephrology)
- Moonlighting (non-nephrology inpatient)
- Moonlighting (emergency department)
- Joint venture with a dialysis provider
- Medical directorship with a dialysis provider
- Basic science research
- Clinical research
- Other

44. Please specify.

45. What is the zip code of the practice address of your primary nephrology job? If unknown or outside the United States please enter city and state/country.

46. Is this practice address located in a federally designated Health Professional Shortage Area?

47. How many years do you expect to be at your primary nephrology job?

48. Which best describes the demographics of the area of your primary nephrology job?

49. How much time do you expect to spend in these areas?

- Direct patient care
- Research
- Teaching
- Administration
- Volunteering/community service

50. In your nephrology practice (primary and secondary jobs) what 3 diseases or conditions do you expect to be involved with most frequently? (Select 3.)

- CKD
- ESRD
- Hypertension
- AKI
- CKD-MBD
- GN
- Diabetic nephropathy

- Kidney transplantation
- Fluid electrolyte and acid-base disorders
- Cystic kidney diseases
- Nephrolithiasis
- Anemia
- Kidney cancer
- In-center conventional hemodialysis
- In-center nocturnal hemodialysis
- Home hemodialysis
- Home peritoneal dialysis

51. Please identify all of the incentives you received for accepting your primary nephrology job. (Select all that apply.)

- H-1 visa sponsorship
- J-1 visa waiver
- Sign-on bonus
- Income guarantees
- On-call payments
- Relocation allowances
- Spouse/partner job transition assistance
- Support for maintenance of certification and continuing medical education
- Career development opportunities
- Educational loan repayment
- None
- Other

52. Please specify.

53. How important were these incentives in your decision to accept your primary nephrology job?

54. What is your expected base salary from your primary nephrology job during your first year of practice?

55. What is your expected additional incentive income from your primary nephrology job during your first year of practice?

56. What is your expected base salary from your secondary nephrology job during your first year of practice?

57. What is your level of satisfaction with your overall salary/compensation?

58. Rank the following factors when selecting a position.

- Predictable start and end time each workday
- Length of each workday
- Frequency of overnight calls
- Frequency of weekend duties
- Job/practice in desired location
- Job/practice in desired practice setting (e.g., hospital group practice, etc.)
- Job/practice meets visa status requirements
- Salary/compensation
- Cost of living
- Cost of establishing a medical practice

- Cost of malpractice insurance
- Taxes
- Employment opportunities for spouse/partner
- Proximity to family
- Climate/weather
- Other factor(s)

59. Please specify other factor(s).

60. What is your overall assessment of nephrology job opportunities within 50 miles of your training site?

61. What is your overall assessment of nephrology job opportunities nationally?

62. Would you recommend nephrology to current medical students and residents?

63. Please explain.

64. Please explain.

65. When did you decide you wanted to pursue nephrology as a specialty?

66. Did you participate in a nephrology rotation during your residency?

67. Did you seriously consider pursuing a career in another area of medicine before you decided on nephrology? (Select all that apply.)

68. Please specify.

69. Which of the following are used in your fellowship curriculum? (Select all that apply.)

- Curriculum Lectures-led by fellows
- Curriculum Lectures-led by attendings
- Journal Clubs-led by fellows
- Journal Clubs-led by attendings
- Fellow-led case reports (morning report style)
- Grand Rounds-presented by fellows (formal presentation on a case/topic)
- Grand Rounds-presented by attendings (formal presentation on a case/topic)
- Key articles or reading lists/collections
- Online audio/video recordings of presentations (e.g., Grand Rounds Lectures)
- Online collaborative learning forum where fellows can post questions cases resources and presentations
- Uninterrupted protected time for renal physiology pathophysiology and/or clinical nephrology review (separate from core-curriculum lectures)
- Renal Pathology conferences
- Morbidity and Mortality conferences
- Kidney biopsy simulation training
- Temporary dialysis catheter simulation training
- Formal ultrasound training
- Interventional nephrology training
- Communications training for end-of-life care and dialysis decision-making

70. Which topics would you most like to receive additional instruction in during fellowship? (Select up to 5.)

- Hemodialysis
- Peritoneal dialysis
- Home hemodialysis
- Temporary dialysis catheter placement
- Kidney biopsy
- Inpatient general AKI diagnosis/management
- Acute GN diagnosis/management
- Nephrotic syndrome
- Diabetic nephropathy
- Outpatient CKD diagnosis/management
- Secondary hypertension diagnosis/management
- Electrolyte disorders/acid-base disorders
- Anemia of renal disease management
- Mineral and bone-disease management
- Nephrolithiasis
- Urinalysis
- Kidney US interpretation
- Renal pathology interpretation
- Post-transplant acute inpatient management
- Post-transplant outpatient management
- Care of adults with pediatric renal disease
- Genetic renal diseases
- Obstetric nephrology
- Toxicology
- Nutrition
- Renal pharmacology
- Geriatric nephrology
- Conservative/palliative management of ESRD
- Other

71. Please specify.

72. How would you rate the overall quality of teaching in your fellowship?

73. Which of the following educational tools have you used in the last 3 months? (Select all that apply.)

- UpToDate
- ASN NephSAP
- ASN KSAP)
- JASN articles
- CJASN articles
- AJKD articles
- Journal articles in general
- AJKD Kidney Core Curriculum
- Textbooks
- ASN Online Geriatrics Curriculum
- ASN Online Dialysis Curriculum
- KDIGO/KDOQI Clinical Practice Guidelines
- Renal Fellow Network Blog
- AJKD Blog
- NephJC (Twitter-based journal club)
- Other

- Watching online recordings of Lectures
- Grand Rounds or Journal Clubs
- Renal Pathology Conferences
- Key article reading list with fellow-authored article summaries
- Problem sets ('homework') on various topics
- Computer simulation exercises of HD/PD cases where you can alter prescriptions and follow results
- Dedicated time for renal physiology, pathophysiology, and/or clinical nephrology review
- Fellow-led in-person case-based pathophysiology/management discussions ('morning report style')
- Fellow-led online case-based pathophysiology/management discussions where fellows share and contribute to cases on a regular basis
- Conferences between local fellowships (e.g., in New England) targeted at fellows
- Case-based debates between specialties (e.g., rheumatology and nephrology fellows)
- Communications training for end-of-life care and dialysis decision-making
- Interventional Nephrology Training
- Simulated catheter placement training
- Simulated renal biopsy

74. Please specify.

Item S2: 2021 ASN Nephrology Fellow Survey

2021 Nephrology Fellow Survey

Start of Block: Consent and Training

Q1 **2021 ASN Nephrology Fellow Survey** Thank you for your interest in the Nephrology Fellow Survey, part of a research study directed by Stephen M. Sozio, MD, MHS, MEHP, in collaboration with the American Society of Nephrology. Please review this important information about your rights and protections to decide if you wish to participate in the study. ***Participation is Voluntary.*** Taking part in this research is entirely voluntary, and you may choose not to participate or to stop participating in the study at any time. ***Study Purpose.*** The purpose of this study is to understand trends in the nephrology workforce and job market. Your answers will be analyzed for scholarly research and for reports to inform future nephrology workforce planning. ***Time Required.*** This survey takes between 10 and 15 minutes, and responses can be saved for completion at a later time. You may skip any questions you do not want to answer, and participation can be stopped at any time. ***Potential Risks of Participation.*** Privacy and confidentiality are usually participants' most important concerns, and are discussed in detail below. ***Privacy and Confidentiality Protections.*** All identifying information (e.g., email and IP addresses) will be separated from your responses immediately after you complete the survey. Your responses will be stored on secure research computer servers, and data files will only be available to authorized members of the research team. All reports and published papers derived from the survey will only report aggregated results. ***Benefits of Participation.*** Your participation will provide important information about the nephrology workforce and job market that will help with future workforce planning. Additionally, ASN will award 2 Grand Prizes of Complimentary BRCU Registration (a $1295 value) and 10 Prizes of Complimentary 1-year ASN Membership upon completion of fellowship (a $395 value) to randomly selected survey respondents. All survey respondents will be entered into the drawing for this award, which will be held immediately after the survey closes. ***Study Approval and Further Information.*** This study has been reviewed and approved by the Johns Hopkins University School of Medicine Institutional Research Board (Study # 00205206). Please contact Stephen M. Sozio, MD, MHS, MEHP, Principal Investigator at ssozio@jhmi.edu or the Office of Human Subjects Research Institutional Research Board at the Johns Hopkins University School of Medicine at 410-955-3008 or jhmeirb@jhmi.edu for further information about your rights as a research participant. ***ELECTRONIC CONSENT:*** Please select your choice below. Clicking on the "Agree" button below indicates that: You have read the above information You voluntarily agree to participate You are at least 18 years of age

○ Agree (1)

○ Do Not Agree (2)

Skip To: End of Survey If 2021 ASN Nephrology Fellow Survey Thank you for your interest in the Nephrology Fellow Survey... = Do Not Agree

Page Break

Q2 **How many years of nephrology training will have you completed at the end of the 2020–2021 academic year?**

- ○ 1 (1)
- ○ 2 (2)
- ○ 3 (3)
- ○ 4 or more (4)

Q3 **What nephrology fellowship are you currently pursuing?**

- ○ Adult Nephrology (1)
- ○ Pediatric Nephrology (2)
- ○ Meds/Peds Nephrology (3)

Display This Question:

If What nephrology fellowship are you currently pursuing? = Adult Nephrology

Q4 **What is your current fellowship type?**

- ○ Clinical Nephrology (1)
- ○ Research (2)
- ○ Transplant Nephrology (3)
- ○ Interventional Nephrology (4)
- ○ Onconephrology (5)
- ○ Nephrology–Critical Care Medicine (6)
- ○ Glomerular Disease (7)
- ○ Home Dialysis (8)
- ○ Other (Please specify your current fellowship type) (9)

Display This Question:

If What is your current fellowship type? = Other (Please specify your current fellowship type)

Q57 Please specify your current fellowship type.

____

Q5 **Where did you attend medical school?**

- ○ United States (4)
- ○ Other country (Please specify) (5)

Display This Question:

If Where did you attend medical school? =

Q58 What country did you attend medical school in?

____

Display This Question:

If Where did you attend medical school? =

Q6
What type of medical school did you attend?

○ Allopathic (1)

○ Osteopathic (2)

Q7 **Where did you complete your residency training?**

▼ I did not complete residency training in the U.S. (1) ... Wyoming (52)

End of Block: Consent and Training

Start of Block: Demographics

Q8 **What is your current gender identity?**

○ Man (1)

○ Woman (2)

○ Genderqueer (3)

○ Gender non-binary (4)

○ Gender non-conforming (5)

○ Another Gender (6)

○ Prefer not to answer (7)

Page Break

Q9 **How old are you?**

▼ Prefer not to answer (1) ... ≥55 (29)

Q10 **What is your current relationship status?**

- ◯ Single (1)
- ◯ Married (2)
- ◯ Partnered (3)
- ◯ Widow/widower (4)
- ◯ Divorced (5)
- ◯ Other (6)
- ◯ Prefer not to answer (7)

Q11 **INCLUDING YOURSELF, how many people currently live in your household that are:**

- ◯ Child: (1) ____
- ◯ School Age: 6–12 Years of Age (2) ____
- ◯ Adolescent: 13–17 Years of Age (3) ____
- ◯ Adult: 18–64 Years of Age (4) ____
- ◯ Older Adult: ≥65 Years of Age (5) ____

Q12 **What is your current citizenship status?**

- U.S. citizen (6)
- Permanent resident (7)
- H-1, H-2, or H-3 visa (temporary worker) (8)
- J-1 or J-2 visa (exchange visitor) (9)
- Other visa (Please specify your "other" visa type) (10)
- Prefer not to answer (11)

Display This Question:

If What is your current citizenship status? = Other visa (Please specify your "other" visa type)

Q59 Please specify your current visa type.

____

Q13 **Are you Hispanic or Latinx?**

- Yes (1)
- No (2)
- Prefer not to answer (3)

Q14 **What is your race? (Select all that apply)**

- ▢ American Indian or Alaska Native (1)
- ▢ Black or African American (2)
- ▢ East Asian (e.g., China, Japan, South Korea, Taiwan) (3)
- ▢ Pacific Islander (4)
- ▢ South Asian (e.g., India, Pakistan, Sri Lanka) (5)
- ▢ Southeast Asian (e.g., Philippines, Vietnam, Singapore) (6)
- ▢ White (7)
- ▢ Other (Please specify) (8)
- ▢ Prefer not to answer (9)

Display This Question:

If What is your race? (Select all that apply) = Other (Please specify)

Q60 Please specify your race.

____

Q15 **What is your current level of educational debt in $1000s? Please estimate to the nearest $1000.**

	Educational Debt ($1000s)
	**0 50 100 150 200 250 300 350 400 450 500**

**1 ()**	

Q16 **When did you decide you wanted to pursue nephrology as a specialty?**

- Before medical school (1)
- During medical school (2)
- During 1st year of residency (3)
- During 2nd or 3rd years of residency (4)
- After practicing in a different area of medicine (5)

Q17 **Which of these ASN programs have you participated in? (Select all that apply)**

- ASN Kidney STARS (1)
- ASN Kidney TREKS (2)
- Ben J. Lipps Research Fellowship Program (3)
- Karen L. Campbell, PhD, Travel Support Program for Fellows (4)

Q18 **Would you recommend nephrology to current medical students and residents?**

- Yes (1)
- No (2)

Display This Question:

If Would you recommend nephrology to current medical students and residents? = Yes

Q19 **Please explain why you would recommend nephrology.**

____

Display This Question:

If Would you recommend nephrology to current medical students and residents? = No

Q20 **Please explain why you would not recommend nephrology.**

____

Q21 **Did you consider pursuing a career in another area of medicine before deciding on nephrology? (Select all that apply)**

- ▢ No, I did not consider another specialty(s) (1)
- ▢ No, I did not consider general practice (2)
- ▢ Yes, I considered another specialty(s) (3)
- ▢ Yes, I considered general practice (internal medicine or pediatrics) (4)

End of Block: Demographics

Start of Block: Future Plans

Q22 **What will you do at the end of the 2020–2021 training year? (Select one)**

- ○ Continue Current Fellowship (9)
- ○ Additional Subspecialty Training or Fellowship (10)
- ○ Private Practice (11)
- ○ Academic Practice (12)
- ○ Internal Medicine Residency (13)
- ○ Undecided (14)
- ○ Other (Please specify) (15)

Display This Question:

If What will you do at the end of the 2020–2021 training year? (Select one) = Other (Please specify)

Q61 **What will you do at the end of the 2020–2021 training year?**

____

Display This Question:

If What will you do at the end of the 2020–2021 training year? (Select one) = Additional Subspecialty Training or Fellowship

Q23 **What additional training are you pursuing? (Select one)**

- Research (1)
- Transplant Nephrology (2)
- Interventional Nephrology (3)
- Onconephrology (4)
- Nephrology–Critical Care Medicine (5)
- Glomerular Disease (6)
- Home Dialysis (7)
- Meds/Peds Nephrology (8)
- Other (Please specify your planned fellowship type) (9)

Display This Question:

If What additional training are you pursuing? (Select one) = Other (Please specify your planned fellowship type)

Q62 Please specify your planned fellowship type.

____

Q24 **Do you plan to stay in the United States for your first post-fellowship job?**

- Yes (1)
- No (2)
- I don't know (3)

Display This Question:

If Do you plan to stay in the United States for your first post-fellowship job? = Yes

Q25 **Do you plan to stay in your current state for your first post-fellowship job?**

○ Yes (3)

○ No (4)

○ I don't know (5)

Display This Question:

If Do you plan to stay in your current state for your first post-fellowship job? = Yes

Q26 **Do you plan to stay in your current city/region for your first post-fellowship job?**

○ Yes (3)

○ No (4)

○ I don't know (5)

Display This Question:

If Do you plan to stay in your current city/region for your first post-fellowship job? = Yes

Q27 **Do you plan to stay in your current institution for your first post-fellowship job?**

- Yes (1)
- No (2)
- I don't know (3)

End of Block: Future Plans

Start of Block: Job Market Experiences

Display This Question:

If How many years of nephrology training will have you completed at the end of the 2020–2021 academi... != 1

And What nephrology fellowship are you currently pursuing? = Adult Nephrology

Or If

How many years of nephrology training will have you completed at the end of the 2020–2021 academi... != 1

Or How many years of nephrology training will have you completed at the end of the 2020–2021 academi... != 2

And What nephrology fellowship are you currently pursuing? = Pediatric Nephrology

Or If

How many years of nephrology training will have you completed at the end of the 2020–2021 academi... != 1

Or How many years of nephrology training will have you completed at the end of the 2020–2021 academi... != 2

And What nephrology fellowship are you currently pursuing? = Meds/Peds Nephrology

Q28
Have you searched for a job beginning after the 2020–2021 training year?

- Yes (1)
- No (2)

Display This Question:

If Have you searched for a job beginning after the 2020–2021 training year? = Yes

Q29 **What types of jobs have you searched for? (Select all that apply)**

- ☐ Clinical Nephrology (1)
- ☐ Clinical Nephrology and Other Clinical Specialty (e.g., critical care) (2)
- ☐ Nephrology—Research (3)
- ☐ Nephrology—Industry (4)
- ☐ Nephrology—Government (5)
- ☐ Non-nephrology—Hospitalist (6)
- ☐ Non-nephrology—Primary care (7)
- ☐ Non-nephrology—Other Clinical Specialty (e.g., critical care) (8)
- ☐ Non-nephrology—Research (9)
- ☐ Non-nephrology—Industry (10)
- ☐ Non-nephrology—Government (11)
- ☐ Other (Please specify) (12)

Display This Question:

If What types of jobs have you searched for? (Select all that apply) = Other (Please specify)

Q63 Please specify what type(s) of jobs you have searched for.

____

Display This Question:

If Have you searched for a job beginning after the 2020–2021 training year? = Yes

Q30 **Have you been offered a job beginning after the 2020–2021 training year?**

- ◯ Yes, and I have accepted an offer (1)
- ◯ Yes, but I am still searching (2)
- ◯ No, I have not yet been offered a job (3)

Display This Question:

If Have you searched for a job beginning after the 2020–2021 training year? = Yes

Q31 **Did you have difficulty finding a nephrology job you were satisfied with?**

- ◯ Yes (1)
- ◯ No (2)

Display This Question:

If Did you have difficulty finding a nephrology job you were satisfied with? = Yes

Q32
Why did you have difficulty? (Select all that apply)

A lack of jobs/practice opportunities:

- ▢ That met visa status requirements (1)
- ▢ In a desired location (2)
- ▢ In a desired practice setting (e.g., hospital, group practice) (3)
- ▢ Offering adequate salary/compensation (4)
- ▢ Offering employment opportunities for spouse/partner (5)
- ▢ Other (Please specify) (6)

Display This Question:

If Why did you have difficulty? (Select all that apply) A lack of jobs/practice opportunities: = Other (Please specify)

Q70 Please specify why you had difficulty.

____

Display This Question:

If Have you searched for a job beginning after the 2020–2021 training year? = Yes

Q33 **How many jobs did you apply for?**

- None (1)
- 1 (2)
- 2 (3)
- 3 (4)
- 4 (5)
- 5 (6)
- 6–9 (7)
- ≥10 (8)

Display This Question:

If Have you searched for a job beginning after the 2020–2021 training year? = Yes

Q34 **How many job offers did you receive?**

- None (1)
- 1 (2)
- 2 (3)
- 3 (4)
- 4 (5)
- 5 (6)
- 6–9 (7)
- ≥10 (8)

End of Block: Job Market Experiences

Start of Block: Job Market Perceptions

Q35 **What is your overall assessment of nephrology job opportunities within 50 miles of your training site?**

- Far too few job opportunities (1)
- Too few job opportunities (2)
- Appropriate number of job opportunities (3)
- Too many job opportunities (4)
- Far too many job opportunities (5)
- I don't know (6)

Q36 **What is your overall assessment of nephrology job opportunities nationally?**

- ◯ Far too few job opportunities (1)
- ◯ Too few job opportunities (2)
- ◯ Appropriate number of job opportunities (3)
- ◯ Too many job opportunities (4)
- ◯ Far too many job opportunities (5)
- ◯ I don't know (6)

End of Block: Job Market Perceptions

Start of Block: Job Characteristics

Display This Question:

If Have you been offered a job beginning after the 2020–2021 training year? = Yes, and I have accepted an offer

Q37 **Which best describes the primary focus of your first post-fellowship job? (Select one)**

- ○ Clinical Nephrology (1)
- ○ Clinical Nephrology and Another Clinical Specialty (e.g., Critical Care) (2)
- ○ Nephrology—Research (3)
- ○ Nephrology—Industry (4)
- ○ Nephrology—Government (5)
- ○ Non-nephrology—Hospitalist (6)
- ○ Non-nephrology—Primary care (7)
- ○ Non-nephrology—Other Clinical Specialty (e.g., Critical Care) (8)
- ○ Non-nephrology—Research (9)
- ○ Non-nephrology—Industry (10)
- ○ Non-nephrology—Government (11)
- ○ Other (Please specify) (12)

Display This Question:

If Which best describes the primary focus of your first post-fellowship job? (Select one) = Other (Please specify)

Q64 Please specify the primary focus of your first post-fellowship job.

____

Display This Question:

If Have you been offered a job beginning after the 2020–2021 training year? = Yes, and I have accepted an offer

Q38 **Which best describes your responsibilities in your first post-fellowship job? (Select all that apply)**

- ☐ Inpatient care (1)
- ☐ Outpatient clinic—CKD (2)
- ☐ Outpatient clinic—Transplant (3)
- ☐ Outpatient dialysis (4)
- ☐ Kidney biopsy (5)
- ☐ Dialysis catheter placement (6)
- ☐ Interventional nephrology (7)
- ☐ Joint venture with a dialysis provider (8)
- ☐ Medical directorship with a dialysis provider (9)
- ☐ Apheresis (10)
- ☐ Basic science research (11)
- ☐ Clinical research (12)
- ☐ Education (13)
- ☐ Other (Please specify) (14)

Display This Question:

If Which best describes your responsibilities in your first post-fellowship job? (Select all that ap... = Other (Please specify)

Q65 Please specify your responsibilities in your first post-fellowship job.

____

End of Block: Job Characteristics

Start of Block: Job Location and Service Requirements

Display This Question:

If Have you been offered a job beginning after the 2020–2021 training year? = Yes, and I have accepted an offer

Q39
Is your first post-fellowship job located in the United States?

- Yes (1)
- No (2)

Display This Question:

If Is your first post-fellowship job located in the United States? = Yes

Q40 **Which state is your first post-fellowship job practice located in?**

▼ Alabama (1) ... Wyoming (51)

Display This Question:

If Is your first post-fellowship job located in the United States? = No

Q41 **What country is your first post-fellowship job located in?**

____

Display This Question:

If Is your first post-fellowship job located in the United States? = Yes

Q42 **Does your first post-fellowship job entail a state, regional, or federal service obligation?**

- Yes (1)
- No (2)

Display This Question:

If Does your first post-fellowship job entail a state, regional, or federal service obligation? = Yes

Q43 **Which best describes the reason for your service obligation?**

- Visa waiver program (1)
- National Health Service Corps or other loan forgiveness program (2)
- Other (Please specify) (3)

Display This Question:

If Which best describes the reason for your service obligation? = Other (Please specify)

Q66 Please specify the reason for your service obligation.

____

Display This Question:

If Is your first post-fellowship job located in the United States? = Yes

Q44 **What is the demographic area of your first post-fellowship job?**

- ○ Large City (≥50,000 population) (1)
- ○ Small City ((2)
- ○ Suburban Area (3)
- ○ Rural Area (4)

End of Block: Job Location and Service Requirements

Start of Block: Remuneration and Incentives

Display This Question:

If Have you been offered a job beginning after the 2020–2021 training year? = Yes, and I have accepted an offer

Q45 **Please select the incentives received for your first post-fellowship job. (Select all that apply)**

- ☐ H-1 visa sponsorship (1)
- ☐ J-1 visa waiver (2)
- ☐ Sign-on bonus (3)
- ☐ Income guarantees (4)
- ☐ Real estate venture (5)
- ☐ On-call payments (6)
- ☐ Relocation allowances (7)
- ☐ Spouse/partner job transition assistance (8)
- ☐ Support for maintenance of certification and continuing medical education (9)
- ☐ Career development opportunities (10)
- ☐ Educational loan repayment (11)
- ☐ Protected time for research/research "start-up" package (12)
- ☐ Other (Please specify) (13)

Display This Question:

If Please select the incentives received for your first post-fellowship job. (Select all that apply) = Other (Please specify)

Q67 Please specify what incentives you received for your first post-fellowship job.

____

Display This Question:

If If Please select the incentives received for your first post-fellowship job. (Select all that apply) q://QID46/SelectedChoicesCount Is Greater Than or Equal to 1

Q46 **How important were incentives in your decision to accept your first post-fellowship job?**

- ◯ Extremely important (1)
- ◯ Very important (2)
- ◯ Moderately important (3)
- ◯ Slightly important (4)
- ◯ Not at all important (5)

Display This Question:

If Have you been offered a job beginning after the 2020–2021 training year? = Yes, and I have accepted an offer

Q47 **What is your expected base salary in your first year of practice (to the nearest $1000).**

	Expected Base Salary ($1000s)
	0 50 100 150 200 250 300 350 400 450 500
**1 ()**	

Display This Question:

If Have you been offered a job beginning after the 2020–2021 training year? = Yes, and I have accepted an offer

Q48
What is your expected additional incentive income in your first year of practice (to the nearest $1000).

	Expected Incentive Income ($1000s)						
	0	10	20	30	40	50	60
1 ()							

Display This Question:

If Have you been offered a job beginning after the 2020–2021 training year? = Yes, and I have accepted an offer

Q49 **How satisfied are you with your overall salary/compensation?**

- Extremely satisfied (1)
- Somewhat satisfied (2)
- Neither satisfied nor dissatisfied (3)
- Somewhat dissatisfied (4)
- Extremely dissatisfied (5)

Q50 **Please rate the importance of the following factors when considering nephrology job opportunities.**

	Extremely Important (1)	Very Important (2)	Moderately Important (3)	Slightly Important (4)	Not Important (5)
Predictable Workday (1)	○	○	○	○	○
Length of Workday (2)	○	○	○	○	○
Overnight Call Frequency (3)	○	○	○	○	○
Weekend Call Frequency (4)	○	○	○	○	○
Desired Location (5)	○	○	○	○	○
Desired Practice Setting (e.g., group practice, hospital) (6)	○	○	○	○	○
Job Meets Visa Requirements (7)	○	○	○	○	○
Salary/Compensation (8)	○	○	○	○	○
Cost of Living (9)	○	○	○	○	○
Cost of Establishing Practice (10)	○	○	○	○	○
Job Opportunities for Spouse/Partner (11)	○	○	○	○	○
Proximity to Family (12)	○	○	○	○	○
Parental Leave Policy (13)	○	○	○	○	○
Protected Time for Research/Academic Pursuits (14)	○	○	○	○	○

Vacation Time (15)	○	○	○	○	○
Opportunities to Attend Meetings (16)	○	○	○	○	○
Potential Mentors at New Job (17)	○	○	○	○	○

End of Block: Remuneration and Incentives

Start of Block: SARS-CoV-2 and Educational Experiences

Q69 What best describes how you currently use telehealth in your fellowship program in the following settings?

	See All Patients Via Telehealth (1)	See Some Patients Via Telehealth (e.g., COVID-19+ or Low Acuity) (2)	Do Not See Patients Via Telehealth (Examinations in Person) (3)	This Experience Put on Hold for Fellows Since Pandemic (4)	NA (Have Not Started This Experience) (5)
Inpatient Consults (1)	○	○	○	○	○
Outpatient Clinic Patients (2)	○	○	○	○	○
Outpatient Dialysis Patients (3)	○	○	○	○	○

Q53 **What is the current format of conferences?**

- Conferences are still in-person but with small groups (1)
- Conferences are online video format (e.g., Zoom, WebEx, Microsoft Teams) (2)
- Conferences are a mixture of in-person small groups and online video (3)
- Conferences have been suspended (4)

Q54 **How would you rate the overall quality of teaching in your fellowship?**

- Excellent (1)
- Good (2)
- Fair (3)
- Poor (4)

Q55 **Which of the following educational tools have you used in the last 3 months?**

- ▢ Textbooks (1)
- ▢ Journal articles in general (2)
- ▢ JASN articles (3)
- ▢ CJASN articles (4)
- ▢ AJKD articles (5)
- ▢ KDIGO/KDOQI clinical practice guidelines (6)
- ▢ ASN NephSAP (7)
- ▢ ASN KSAP (8)
- ▢ UpToDate (9)
- ▢ Renal Fellow Network (10)
- ▢ Twitter (11)
- ▢ Sermo (12)
- ▢ Doximity (13)
- ▢ NephJC (14)
- ▢ General medicine–focused podcasts (15)

- ▢ Nephrology–focused podcasts (16)
- ▢ NephSIM (17)
- ▢ NephMadness (18)
- ▢ AJKD blog (19)
- ▢ NephroPOCUS (20)
- ▢ GlomCon (21)
- ▢ Arkana Pathology Series (22)

Carry Forward Selected Choices from "Which of the following educational tools have you used in the last 3 months?"

X→

Q57 **Please rate the educational effectiveness of the tools you identified.**

	Very Effective (1)	Effective (2)	Neither Effective nor Ineffective (3)	Ineffective (4)	Very Ineffective (5)
Textbooks (x1)	○	○	○	○	○
Journal articles in general (x2)	○	○	○	○	○
JASN articles (x3)	○	○	○	○	○
CJASN articles (x4)	○	○	○	○	○
AJKD articles (x5)	○	○	○	○	○
KDIGO/KDOQI clinical practice guidelines (x6)	○	○	○	○	○
ASN NephSAP (x7)	○	○	○	○	○
ASN KSAP (x8)	○	○	○	○	○
UpToDate (x9)	○	○	○	○	○
Renal Fellow Network (x10)	○	○	○	○	○
Twitter (x11)	○	○	○	○	○
Sermo (x12)	○	○	○	○	○
Doximity (x13)	○	○	○	○	○
NephJC (x14)	○	○	○	○	○

General medicine–focused podcasts (x15)	○	○	○	○	○
Nephrology–focused podcasts (x16)	○	○	○	○	○
NephSIM (x17)	○	○	○	○	○
NephMadness (x18)	○	○	○	○	○
AJKD blog (x19)	○	○	○	○	○
NephroPOCUS (x20)	○	○	○	○	○
GlomCon (x21)	○	○	○	○	○
Arkana Pathology Series (x22)	○	○	○	○	○

Q58 **Indicate your agreement with the following statements about your educational and professional development.**

	Strongly Agree (1)	Agree (2)	Neither Agree nor Disagree (3)	Disagree (4)	Strongly Disagree (5)
During the pandemic my fellowship training program has been successful in sustaining my education. (1)	○	○	○	○	○
I will be adequately prepared for independent general nephrology practice upon completion of fellowship. (2)	○	○	○	○	○
There is a good sense of community within my training program. (3)	○	○	○	○	○
I have good relationships with my mentors. (4)	○	○	○	○	○
I am provided the support to complete/advance my research. (5)	○	○	○	○	○
I have been exposed to a wide variety of clinical experiences. (6)	○	○	○	○	○
I have adequate time to prepare for board certification. (7)	○	○	○	○	○
I am satisfied with the career mentorship I have received during fellowship training. (8)	○	○	○	○	○

I am satisfied with the advice I received on finding and negotiating salary and expectations in my first post-fellowship job. (9)	o	o	o	o	o
I am satisfied with my work-life balance. (10)	o	o	o	o	o
I am satisfied with nephrology as a career. (11)	o	o	o	o	o

End of Block: SARS-CoV-2 and Educational Experiences

Table S1. 2021 Fellow Survey Respondent Demographics and Comparison with ACGME Data 2020–2021*

Variable	Adult Nephrology		Pediatric Nephrology		Adult/Pediatric Nephrology
	Respondents	ACGME	Respondents	ACGME	Respondents
Educational Status †					
USMG	214 (46%)	285 (34%)	14 (45%)	87 (73%)	3 (60%)
IMG	251 (54%)	543 (66%)	17 (55%)	33 (27%)	2 (40%)
Years of Training Completed ‡					
1	206 (44%)	416 (50%)	5 (16%)	49 (41%)	1 (20%)
2	238 (51%)	413 (50%)	15 (48%)	32 (57%)	2 (40%)
3	11 (2%)	—	11 (35%)	39 (33%)	—
4 or more	10 (2%)	—	—	—	2 (40%)
Gender Identity §					
Man	270 (58%)	500 (60%)	8 (26%)	23 (19%)	1 (20%)
Woman	189 (41%)	317 (38%)	23 (74%)	96 (80%)	4 (80%)
Prefer not to answer	4 (1%)	12 (1%)	—	1 (1%)	—
Citizenship Status					
U.S. citizen	257 (56%)	—	16 (52%)	—	5 (100%)
Permanent resident	45 (10%)	—	3 (10%)	—	—
H-1, H-2, or H-3 visa (temporary worker)	41 (9%)	—	2 (6%)	—	—
J-1 or J-2 visa (exchange visitor)	103 (22%)	—	9 (29%)	—	—
Other visa (Please specify	2 (0%)	—	1 (3%)	—	—

your "other" visa type)					
Prefer not to answer	13 (3%)	—	—	—	—
Ethnicity					
Hispanic/Latinx	37 (8%)	72 (9%)	3 (10%)	0	1 (20%)
Prefer not to answer	11 (2%)	—	—	—	—
Race					
American Indian or Alaska Native	3 (0.6%)	0	0 (0.0%)	0	0 (0.0%)
Black or African American	24 (5.1%)	40 (5%)	2 (6.5%)	6 (5%)	0 (0.0%)
East Asian (e.g., China, Japan, South Korea, Taiwan)	38 (8.1%)	370 (45%)	3 (9.7%)	30 (25%)	0 (0.0%)
Pacific Islander	0 (0.0%)	0	0 (0.0%)	0	0 (0.0%)
South Asian (e.g., India, Pakistan, Sri Lanka)	151 (32.2%)	—	7 (22.6%)	—	0 (0.0%)
Southeast Asian (e.g., Philippines, Vietnam, Singapore)	28 (6.0%)	—	1 (3.2%)	—	1 (20.0%)
White	162 (34.5%)	239 (29%)	15 (48.4%)	65 (54%)	3 (60.0%)
Other (Please specify)	36 (7.7%)	33 (4%)	2 (6.5%)	3 (3%)	0 (0.0%)
Prefer not to answer	27 (5.8%)	34 (4%)	1 (3.2%)	4 (3%)	1 (20.0%)
Census Division					
East North Central	58 (13%)	—	4 (14%)	—	1 (20%)
East South Central	21 (5%)	—	3 (10%)	—	—

Middle Atlantic	96 (22%)	—	6 (21%)	—	1 (20%)
Mountain	18 (4%)	—	—	—	—
New England	39 (9%)	—	—	—	—
Pacific	58 (13%)	—	4 (14%)	—	—
South Atlantic	76 (18%)	—	7 (24%)	—	2 (40%)
West North Central	29 (7%)	—	1 (3%)	—	
West South Central	36 (8%)	—	4 (14%)	—	1 (20%)

*Accreditation Council for Graduate Medical Education. *Data Resource Book Academic Year 2020-2021.* Chicago, IL: Accreditation Council for Graduate Medical Education; 2022.
†Goodness of fit comparison between participants and 2020–2021 ACGME data—adult respondents, $p<0.05$; pediatric respondents, $p<0.05$; Chi-squared test.
‡Goodness of fit comparison between participants and 2020–2021 ACGME data—adult respondents, p=0.22; pediatric respondents, p=0.02; Chi-squared test.
§Goodness of fit comparison between participants and 2020–2021 ACGME data—adult respondents, p=0.47; pediatric respondents, p=0.59; Chi-squared test.
